# Surface Reconstruction from Parallel Curves with Application to Parietal Bone Fracture Reconstruction

**DOI:** 10.1371/journal.pone.0149921

**Published:** 2016-03-11

**Authors:** Abdul Majeed, Abd Rahni Mt Piah, Zainor Ridzuan Yahya

**Affiliations:** 1 Division of Science and Technology, University of Education, Town Ship Lahore, Pakistan; 2 School of Mathematical Sciences, Universiti Sains Malaysia, 11800 Penang, Malaysia; 3 Institute of Engineering Mathematics, Universiti Malaysia Perlis, Pauh Putra Campus, 02600 Pauh, Perlis, Malaysia; University of Zaragoza, SPAIN

## Abstract

Maxillofacial trauma are common, secondary to road traffic accident, sports injury, falls and require sophisticated radiological imaging to precisely diagnose. A direct surgical reconstruction is complex and require clinical expertise. Bio-modelling helps in reconstructing surface model from 2D contours. In this manuscript we have constructed the 3D surface using 2D Computerized Tomography (CT) scan contours. The fracture part of the cranial vault are reconstructed using *GC*^1^ rational cubic Ball curve with three free parameters, later the 2D contours are flipped into 3D with equidistant *z* component. The constructed surface is represented by contours blending interpolant. At the end of this manuscript a case report of parietal bone fracture is also illustrated by employing this method with a Graphical User Interface (GUI) illustration.

## Introduction

**Fig 1:**
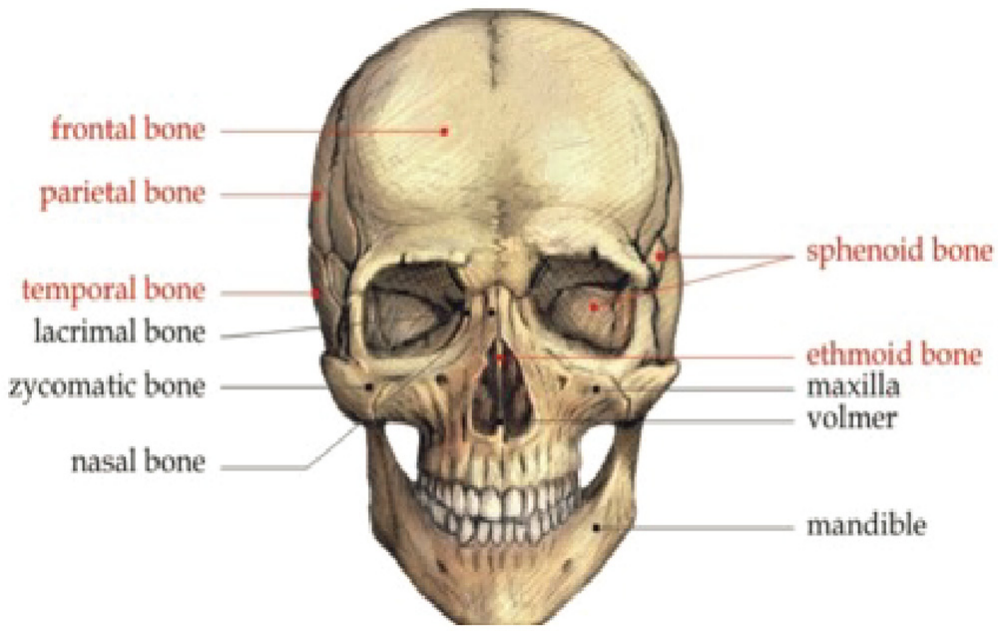
Craniofacial bones.

Craniofacial region is a complex anatomical part made up of various bones joined together. Fig 1 illustrates the various bones that make up the human skull. Craniofacial fractures occurs due to various etiological factors like road traffic accident (RTA), sports injury falls etc. Various diagnostic tools like X rays, Computerized Tomography (CT) scans, Magnetic Resonance Image (MRI) have been used to diagnose the craniofacial fractures. Since, craniofacial fractures do not follow a specific pattern and lately, emerging virtual reconstruction technologies opened new avenues for mathematicians, physicists and software engineers to reconstruct the fracture defects. Already established approaches for implant design is Computer Aided Design (CAD)/Computer Aided Manufacturing (CAM) process chain [1]. Other alternative methods that design implant without CAD process are mirroring [2] and surface interpolation also called as deformation [3].

The methods used for the construction of fracture segment are either anatomical or mathematical features. The mirroring method is useful for the fracture on one side of the skull only and use anatomical features. While mathematical constraints are used in interpolation and deformation methods to construct the fractured part from the non fractured part of skull. Reference model is also used to construct the fractured part of skull [4]. For more related work (see [5–23] and references therein).

Contours blending function have been used to construct the 3D parietal bone fracture. Since Digital Imaging and Communications in Medicine (DICOM) data used in our work are in 2D form, so, before constructing the 3D surface, firstly, we have constructed fracture part curves using geometric continuity (*GC*^1^) rational cubic Ball curves with three free parameters. Genetic Algorithm (GA) is used to optimize the free parameters. Then, we converted the 2D contour curves in 3D form by taking equidistant *z* component and then construct the 3D parietal bone fracture.

We present a case report of parietal bone fracture to show the applicability of proposed algorithm. The Dicom data are obtained from Hospital Universiti Sains Malaysia (HUSM). We employed matlab for both the programming and to develop Graphical User Interface (GUI) for parietal bone fracture reconstruction using proposed algorithm. Surgeons can use GUI for fracture reconstruction without having in depth knowledge of its mathematical aspect.

The introduction is followed by the representation of Ball basis functions and rational cubic Ball curve. It is followed by an explanation on contour blending, boundary extraction, parametrization, Normalized mean squares error. After this, we have explained the Graphical User Interface (GUI). An algorithm on 3D parietal bone fracture reconstruction using rational Ball curve and contour blending is proposed after the explanation of GUI.

## Cubic Ball Basis Functions and Curve

**Fig 2:**
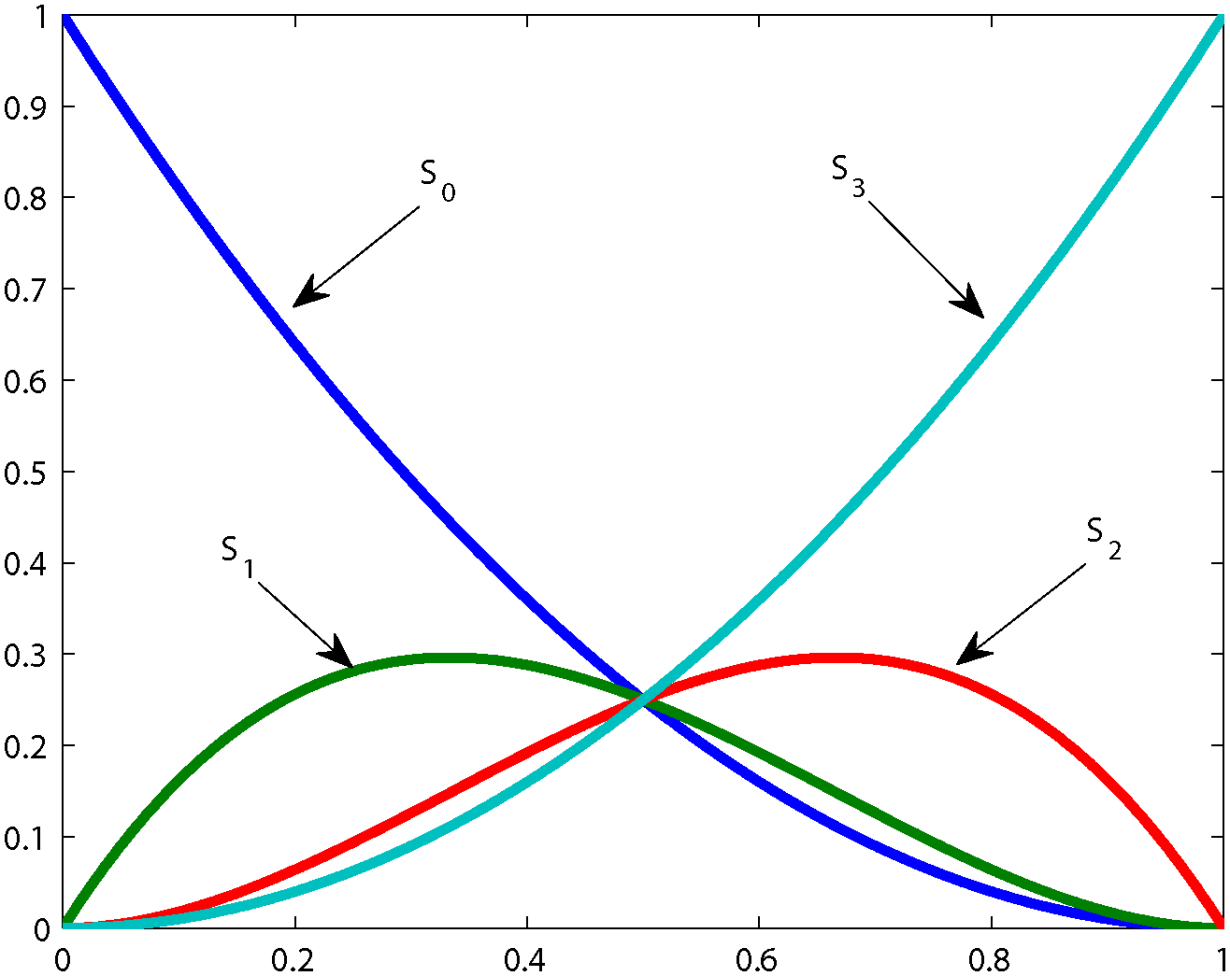
Ball basis functions.

The cubic Ball polynomial basis was first proposed by Ball [24] for CAD systems application. Fig 2 illustrates these functions against its parameter *θ*. The Ball basis functions can be written as

$$\begin{array}{c} \begin{array}{c} S_{0}\left(\theta \right)={\left(1-\theta \right)}^{2},\;S_{1}\left(\theta \right)=2\theta {\left(1-\theta \right)}^{2},\\ S_{2}\left(\theta \right)=2\theta^{2}\left(1-\theta \right),\;S_{3}\left(\theta \right)=\theta^{2}. \end{array} \end{array}$$ (1)

**Theorem 1:** The Ball basis functions defined in Eq (1) have the following properties

- Linearly Independent: The Ball basis functions are linearly independent. There do not exist set of non zero constants *a*_0_, …, *a*_3_, for which.
  $$\begin{array}{c} \sum_{i=0}^{3}a_{i}S_{i}\left(\theta \right)=0. \end{array}$$
- Non Negative: Ball basis functions are non negative when *θ* ∈ [0, 1].
- Symmetric: The Ball basis functions are symmetric as
  $$\begin{array}{c} S_{i}\left(\theta \right)=S_{3-i}\left(1-\theta \right). \end{array}$$
- Monotonicity: *S*_0_(*θ*) is monotonically decreasing and *S*_3_(*θ*) is monotonically increasing when *θ* ∈ [0, 1].
- Partition of Unity: Ball basis functions form a partition of unity, it means that sum of Ball basis functions will be 1.
  $$\begin{array}{c} \sum_{i=0}^{3}S_{i}\left(\theta \right)=1. \end{array}$$

**Theorem 2:** Let *P*_*i*_ be the set of control points and *S*_*i*_(*θ*), *i* = 0, …, 3 are Ball basis functions defined in Eq (1). The Ball curve $s\left(\theta \right)=\sum_{i=0}^{3}P_{i}S_{i}\left(\theta \right)$ have the following properties

**Fig 3:**
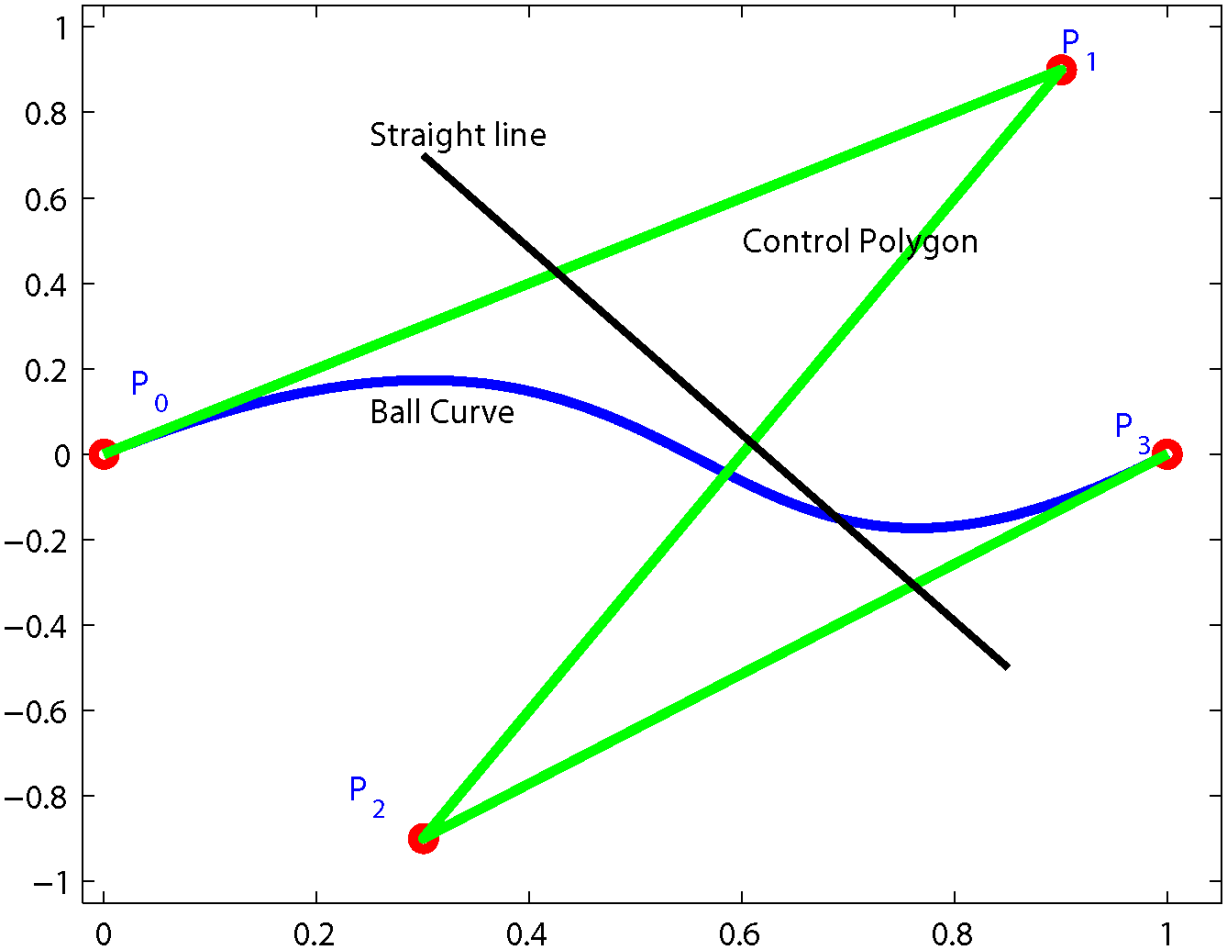
Variation diminishing property with b = 1 p = 3 k = 1.

- Coordinate system independence: As the Ball basis functions form partition of unity, so the Ball basis curve will be coordinate system independence. It means that by changing the coordinate system of control points curve will remain same.
- Convex Hull Property: As the Ball curve obeys the coordinate system independence and Ball basis functions are all non negative. So Ball curves will obey the convex hull property. It means that curves formed by Ball basis always lie within the convex hull of their control points.
  $$\begin{array}{c} \sum_{i=0}^{3}S_{i}\left(\theta \right)=1,\;\;\;S_{i}\left(\theta \right)\geq 0,\;\;0\leq \theta \leq 1,\;i=0,...,3. \end{array}$$
- Variation Diminishing Property (VDP): Variation Dimension Property is obeyed by Ball curves. VDP is stated as if a curve is intercepted by straight line in *b* number of points and control polygon by *p* number of points, then it will always hold that
  $$\begin{array}{c} b=p-2k \end{array}$$
  as shown in Fig 3. Where *k* is 0 or a positive integer.
- Endpoint Interpolation: The Ball curves always passes through the first and last control points.
  $$s(0)=P_{0},\;s(1)=P_{3}.$$

**Theorem 3:** Let *P*_*i*_ be the set of control points and *S*_*i*_(*θ*), *i* = 0, …, 3 are Ball basis functions defined in Eq (1). The Ball curve $s\left(\theta \right)=\sum_{i=0}^{3}P_{i}S_{i}\left(\theta \right)$ have the following distinctive advantages over Bezier basis curve

**Fig 4:**
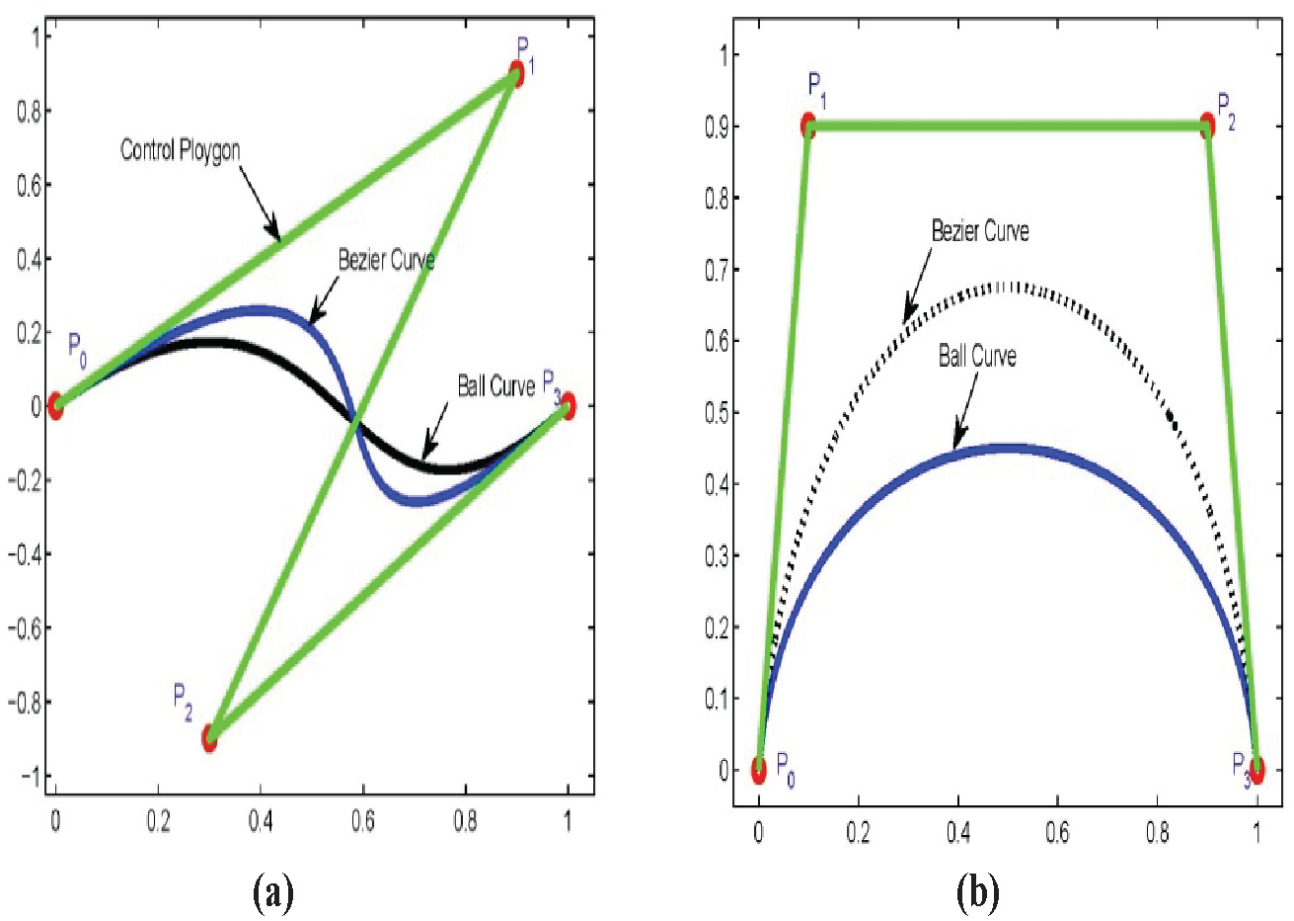
Ball verses Bezier curve.

- The cubic Ball curve reduce to quadratic curve if *P*_1_ = *P*_2_.
  $$\begin{array}{c} \begin{array}{c} s\left(\theta \right)=S_{0}\left(\theta \right)P_{0}+2S_{1}\left(\theta \right)P_{1}+2S_{2}\left(\theta \right)P_{2}+S_{3}\left(\theta \right)P_{3},\\ ={\left(1-\theta \right)}^{2}P_{0}+2\theta {\left(1-\theta \right)}^{2}P_{1}+2\theta^{2}\left(1-\theta \right)P_{1}+\theta^{2}P_{3},\\ ={\left(1-\theta \right)}^{2}P_{0}+2\theta \left(1-\theta \right)P_{1}+\theta^{2}P_{3}. \end{array} \end{array}$$
- The Ball curve passed away from the convex hull intermediate control points as compared to the Bezier curve as shown in Fig 4.
- Operation of degree elevation and reduction for a polynomial curve with Ball basis functions are simpler and faster than the curve with Bezier basis functions [25].

## *GC*^1^ Rational Cubic Ball Curve

The cubic rational Ball curve *s*(*θ*) defined in [26, 27] is

$$\begin{array}{c} s\left(\theta \right)=\frac{P(\theta )}{Q(\theta )},\;0\leq \theta \leq 1, \end{array}$$ (2)

where

$$\begin{array}{c} \begin{array}{c} P\left(\theta \right)=A{\left(1-\theta \right)}^{2}+B{\left(1-\theta \right)}^{2}\theta +C\left(1-\theta \right)\theta^{2}+D\theta^{2},\\ Q\left(\theta \right)={\left(1-\theta \right)}^{2}+a{\left(1-\theta \right)}^{2}\theta +b\left(1-\theta \right)\theta^{2}+\theta^{2}. \end{array} \end{array}$$

For *GC*^1^ continuity consider the two consecutive curve segments

$$\begin{array}{c} s_{i}\left(\theta \right)=\frac{P_{i}\left(\theta \right)}{Q_{i}\left(\theta \right)},\;i=1,...,n-1, \end{array}$$ (3)

$$\begin{array}{c} s_{i+1}\left(\theta \right)=\frac{P_{i+1}\left(\theta \right)}{Q_{i+1}\left(\theta \right)},\;i=1,...,n-1. \end{array}$$ (4)

where

$$\begin{array}{c} \begin{array}{c} P_{i}\left(\theta \right)=A_{i}{\left(1-\theta \right)}^{2}+B_{i}{\left(1-\theta \right)}^{2}\theta +C_{i}\left(1-\theta \right)\theta^{2}+D_{i}\theta^{2},\\ Q_{i}\left(\theta \right)={\left(1-\theta \right)}^{2}+a_{i}{\left(1-\theta \right)}^{2}\theta +b_{i}\left(1-\theta \right)\theta^{2}+\theta^{2},\\ P_{i+1}\left(\theta \right)=A_{i+1}{\left(1-\theta \right)}^{2}+B_{i+1}{\left(1-\theta \right)}^{2}\theta +C_{i+1}\left(1-\theta \right)\theta^{2}+D_{i+1}\theta^{2},\\ Q_{i+1}\left(\theta \right)={\left(1-\theta \right)}^{2}+a_{i+1}{\left(1-\theta \right)}^{2}\theta +b_{i+1}\left(1-\theta \right)\theta^{2}+\theta^{2}. \end{array} \end{array}$$

satisfying the following conditions

$$\begin{array}{c} \left\{\begin{array}{c} s_{i}\left(0\right)=A_{i},\;s_{i}\left(1\right)=D_{i},\\ s_{i+1}\left(0\right)=A_{i+1},\;s_{i+1}\left(1\right)=D_{i+1},\\ s_{i}\left(1\right)=s_{i+1}\left(0\right),\\ s_{i+1}^{\prime }\left(0\right)=\lambda_{i}s_{i}^{\prime }\left(1\right). \end{array}\right. \end{array}$$ (5)

using the above geometric continuity condition we can write

$$\begin{array}{c} \left\{\begin{array}{c} s_{i}\left(0\right)=f_{i}=A_{i},\;s_{i}\left(1\right)=f_{i+1},\\ s_{i+1}\left(0\right)=f_{i+1},\;s_{i+1}\left(1\right)=f_{i+2},\\ D_{i}=A_{i+1}=f_{i+1},\\ B_{i+1}=\left(a_{i+1}+\lambda_{i}b_{i}\right)f_{i+1}-\lambda_{i}C_{i}. \end{array}\right. \end{array}$$ (6)

where *f*_*i*_, *f*_*i* + 1_, *f*_*i* + 2_ *i* = 1, …, *n* − 1 are the on curve control points and *a*_*i*_, *b*_*i*_ and *λ*_*i*_ are free parameters. For curve fitting, firstly, we will find off curve control points using least square method. Then, free parameters will be optimized with the help of genetic algorithm.

In [28] the continuity between two curve segments is parametric continuity *C*^1^ while in current work the continuity between two curve segments is geometric continuity *GC*^1^ as the geometric continuity is more flexible than parametric. We have used *C*^1^ rational Ball curves with tangents at end points. The tangent vectors work as intermediate control points, while in current work we are using *GC*^1^ rational Ball curves and the intermediate control points are evaluated using least square method. In the current work the number of free parameters is increased to three and the proposed method is more flexible due to its geometric continuity. The proposed method works well for both small and large fractured parts. In current work we have extended the 2D CT scan data into 3D format taking equidistant *z*-component to construct the 3D craniofacial fractured part.

**Effect of Control Point and Free Parameters**

**Fig 5:**
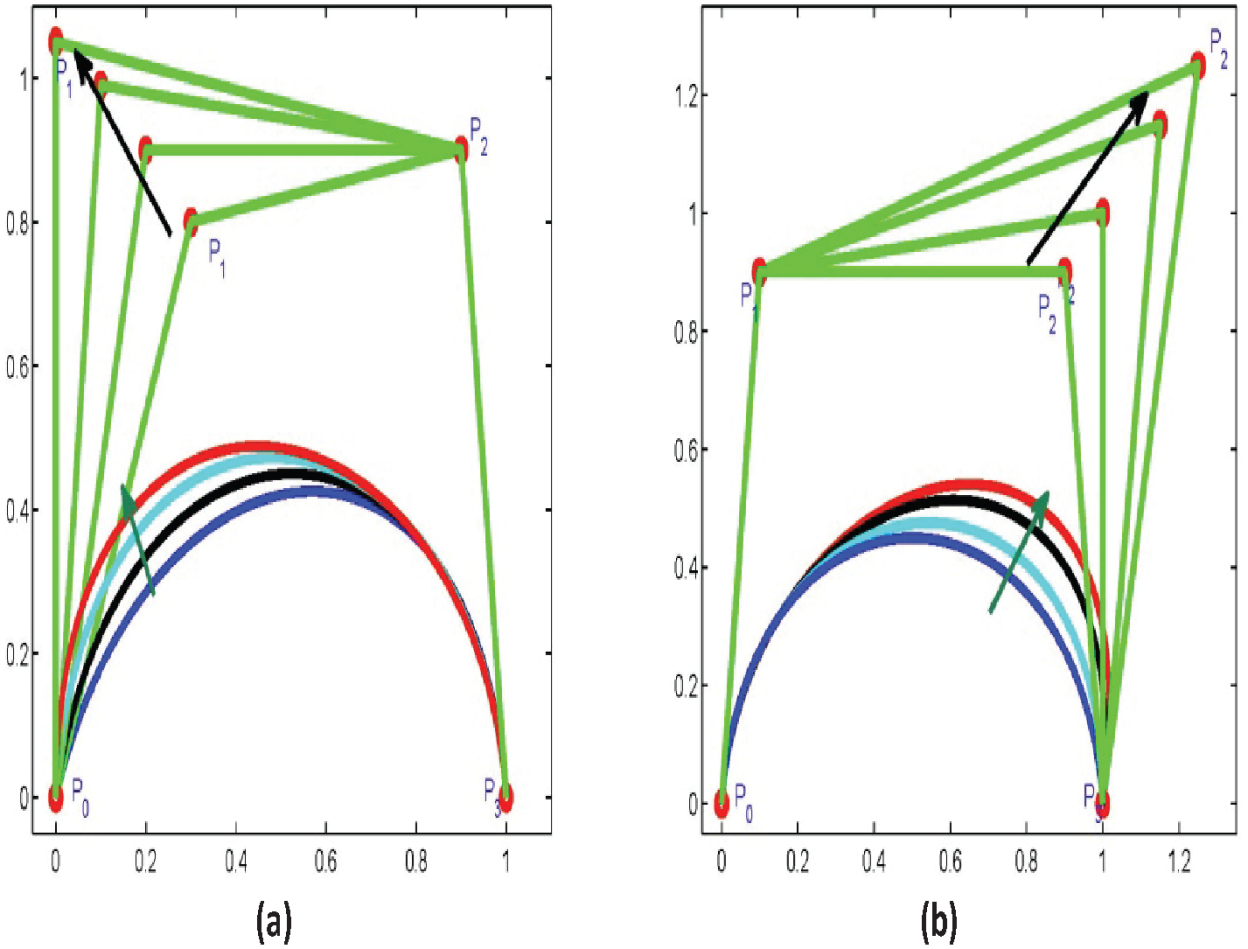
Effect of control points on curve.

**Fig 6:**
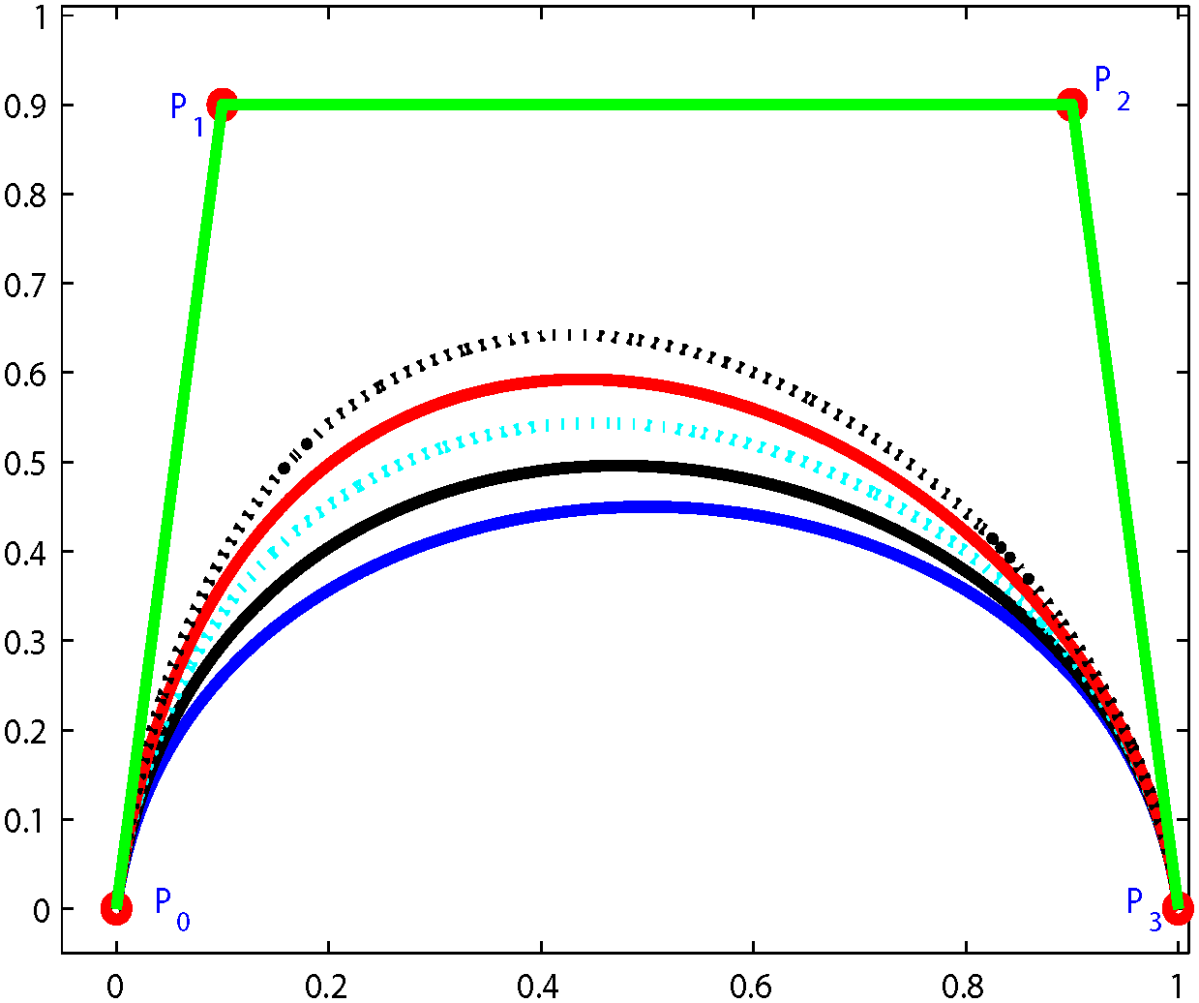
Effect of free parameters black curve when a = 1, b = 1 blue a = 1.4, b = 1 black a = 1.6, b = 1 cyan a = 1.8, b = 1 red a = 2, b = 1 black.

Ball curve can be changed or controlled by twofold. Firstly, by changing the off curve control points. For example in Fig 5, by changing the positron of *P*_1_ control point the curve bend toward *P*_1_. Similarly, with *P*_2_ control point keeping the free parameters unchanged. Secondly, by changing the free parameter *a* and keeping *b* constant. For example in Fig 6, the curve bends toward *P*_1_. Likewise, by changing *b* the curve will bend toward *P*_2_.

The given Dicom data are in 2*D* form. To construct the surface, patch one have to convert the data in 3*D* form. For this, we took the non decreasing *z* component of each 2*D* contours as a height like *z*_0_ < *z*_1_ < … < *z*_*max*_. The *i*^*th*^ contour is defined by the sequence of distinct data points which are counterclockwise ordered in the contour at the height *z*_*i*_.

### Contours Blending

The fracture part curves for each 2*D* contour have been constructed using proposed interpolant, and is converted into 3*D* form taking equidistant *z* component [29]. Let *C*^*i* − 1^(*ψ*), *C*^*i*^(*ψ*) and *C*^*i* + 1^(*ψ*) are missing part curves at height *z*^*i* − 1^, *z*^*i*^ and *z*^*i* + 1^ respectively. The cubic Ball interpolant can be written as

$$\begin{array}{c} B^{i}\left(\theta ,\psi \right)=C^{i}\left(\psi \right)S_{0}\left(\theta \right)+\left(z^{i+1}-z^{i}\right)v^{i}\left(\psi \right)S_{1}\left(\theta \right)+v^{i+1}\left(\psi \right)S_{2}\left(\theta \right)+C^{i+1}\left(\psi \right)S_{3}\left(\theta \right), \end{array}$$ (7)

for *i* = 1, …, *n* − 1, 0 ≤ *θ*, *ψ* ≤ 1,

where *n* represents the number of contours. *S*_*j*_, *j* = 0, …, 3. are Ball basis polynomial functions defined in Eq (1).

*v*^*i*^(*ψ*) is a tangent to surface at (*C*^*i*^(*ψ*), *z*^*i*^), to define the tangent vector let

*P*^*i*^(*ψ*) = (*C*^*i*^(*ψ*), *z*^*i*^) for *i* = 1, …, *n*. Where *P*^*i*^(*ψ*) is in 3D form because *C*^*i*^(*ψ*) is in 2*D* form. Let

$$\begin{array}{c} {\hat{V}}_{i}\left(\psi \right)=|C^{i+1}\left(\psi \right)-C^{i}\left(\psi \right)|\left(P^{i}\left(\psi \right)-P^{i-1}\left(\psi \right)\right)+\left|C^{i}\left(\psi \right)-C^{i-1}\left(\psi \right)\right|\left(P^{i+1}\left(\psi \right)-P^{i}\left(\psi \right)\right). \end{array}$$

Thus ${\hat{V}}_{i}\left(\psi \right)$ is a complex combination of the vector (*P*^*i*^(*ψ*) − *P*^*i* − 1^(*ψ*)) and (*P*^*i* + 1^(*ψ*) − *P*^*i*^(*ψ*)). The *z* − *component* of ${\hat{V}}_{i}\left(\psi \right)$ is

|*C*^*i* + 1^(*ψ*) − *C*^*i*^(*ψ*)|(*z*^*i*^ − *z*^*i* − 1^) + |*C*^*i*^(*ψ*) − *C*^*i* − 1^(*ψ*)|(*z*^*i* + 1^ − *z*^*i*^). Let *V*_*i*_(*ψ*) be the vector product when ${\hat{V}}_{i}\left(\psi \right)$ is divided by its *z* − *component*.

Then *V*_*i*_(*ψ*) = (*v*^*i*^(*ψ*), 1) where

$$\begin{array}{c} v^{i}\left(\psi \right)=\frac{|C^{i+1}\left(\psi \right)-C^{i}\left(\psi \right)|\left(C^{i}\left(\psi \right)-C^{i-1}\left(\psi \right)\right)+\left|C^{i}\left(\psi \right)-C^{i-1}\left(\psi \right)\right|\left(C^{i+1}\left(\psi \right)-C^{i}\left(\psi \right)\right)}{|C^{i+1}\left(\psi \right)-C^{i}\left(\psi \right)|\left(z^{i}-z^{i-1}\right)+\left|C^{i}\left(\psi \right)-C^{i-1}\left(\psi \right)\right|\left(z^{i+1}-z^{i}\right)}. \end{array}$$

$$\begin{array}{c} v^{i}\left(\psi \right)=\frac{|D_{1}|D_{2}+\left|D_{2}\right|D_{1}}{D_{1}\left(z^{i}-z^{i-1}\right)+D_{2}\left(z^{i+1}-z^{i}\right)} \end{array}$$

where *D*_1_ = *C*^*i* + 1^(*ψ*) − *C*^*i*^(*ψ*) and *D*_2_ = *C*^*i*^(*ψ*) − *C*^*i* − 1^(*ψ*).

The vector *V*_*i*_(*ψ*) will be chosen to be a tangent to the surface at *P*^*i*^(*ψ*). If *C*^*i* + 1^(*ψ*) = *C*^*i*^(*ψ*) = *C*^*i* − 1^(*ψ*), then *D*_1_ = *D*_2_ = 0 and *v*^*i*^(*ψ*) is set to be (0, 0).

As *v*_*i*_(*ψ*) lies in a plane containing *P*^*i* + 1^(*ψ*), *P*^*i*^(*ψ*) and *P*^*i* − 1^(*ψ*). If these points lie in a vertical plane, then

$$\begin{array}{c} C^{i+1}\left(\psi \right)-C^{i}\left(\psi \right)=\lambda v^{*}\left(\psi \right) \end{array}$$

$$\begin{array}{c} C^{i}\left(\psi \right)-C^{i-1}\left(\psi \right)=\mu v^{*}\left(\psi \right) \end{array}$$

for some unit vector v*(*ψ*) and some scalers *λ* and *μ*. Then

$$\begin{array}{c} v^{i}\left(\psi \right)=\frac{|\mu |\lambda +|\lambda |\mu }{\left|\mu \right|\left(z^{i+1}-z^{i}\right)+\left|\lambda \right|\left(z^{i}-z^{i-1}\right)}v^{*}\left(\psi \right) \end{array}$$

If *λμ* < 0, then |*μ*|*λ* = −|*λ*|*μ* and *v*^*i*^(*ψ*) = 0.

If *λμ* > 0, then

$$\begin{array}{c} v^{i}\left(\psi \right)=\frac{2\mu \lambda }{\mu \left(z^{i+1}-z^{i}\right)+\lambda \left(z^{i}-z^{i-1}\right)}v^{*}\left(\psi \right) \end{array}$$

Dividing denominator and numerator by (*z*^*i* + 1^ − *z*^*i*^)(*z*^*i*^ − *z*^*i* − 1^),

$$\begin{array}{c} v^{i}\left(\psi \right)=\frac{\frac{2\mu \lambda }{\left(z^{i+1}-z^{i}\right)\left(z^{i}-z^{i-1}\right)}}{\frac{\mu }{\left(z^{i}-z^{i-1}\right)}+\frac{\lambda }{\left(z^{i+1}-z^{i}\right)}}v^{*}\left(\psi \right) \end{array}$$ (8)

This definition for *v*^*i*^(*ψ*) is proposed by [30] for a comonotone univariate interpolation scheme.

Finally the surface between two consecutive contour *z*^*i*^ and *z*^*i* + 1^ is given by,

$$\begin{array}{c} S^{i}\left(\psi ,\theta \right)=\left(B^{i}\left(\theta ,\psi \right),\left(1-\theta \right)z^{i}+\theta z^{i+1}\right) \end{array}$$ (9)

Now the surface between *z*^1^ and *z*^*n*^ is

$$\begin{array}{c} S\left(\psi ,\theta \right)=\left\{S^{i}\left(\psi ,\theta \right);\;i=1,...,n-1,\;0⩽\psi ,\theta ⩽1\right\} \end{array}$$ (10)

### Boundary Extraction and Corner Detection

To construct the parietal bone fracture, we will initially determined the boundary of each CT scan image using mathematical morphology. The mathematical morphology is defined as *β*(*A*) = *A* − (*A*Θ*B*). In this equation *A* represents the set of all black pixels, *B* represents 3 × 3 structured elements, *β*(*A*) is the boundary set of *A*, − and Θ represents the difference and erosion operator. To divide the boundary in smaller segments, we use the corner points. Sarfarz et al method [31] is employed to find the corner points.

### Parameterization

This manuscript employs the chord length parametrization to find the values of *θ*_*i*_ corresponding to *D*_*i*_, where *D*_*i*_ represents the data points of each segments.

$$\begin{array}{c} \left\{\begin{array}{c} \theta_{0}=0,\\ \theta_{k}=\frac{\sum_{i=1}^{k}\left|D_{i}-D_{i+1}\right|}{\sum_{i=1}^{n}\left|D_{i}-D_{i+1}\right|}\;1\leq k\leq n-1,\\ \theta_{n}=1. \end{array}\right. \end{array}$$ (11)

### Normalized Mean Squares Error

$$\begin{array}{c} E^{2}=\frac{\sum |s_{i}\left(\theta \right)-D_{i}{|}^{2}}{\sum |D_{i}{|}^{2}}. \end{array}$$ (12)

*D*_*i*_ are the data points of each segments and *θ* is parameterized by chord length. We will find the free parameters *a*_*i*_, *b*_*i*_ *and* *λ*_*i*_ in proposed interpolant by minimizing the cost function. Genetic Algorithm (GA) defined in [28] is used to find the *a*_*i*_, *b*_*i*_ *and* *λ*_*i*_ respectively.

### Graphical User Interface(GUI)

GUI consists of one or more windows having control, these are called components. GUI is utilized to perform the interactive tasks and display it graphically. GUI facilitates the users in completion of different tasks. Users are not required to learn the basic programming of each component. GUI consist of start and stop button, panel, scroll bar, push button and boxes etc.

GUI with its individual controls has a executable MATLAB code known as callbacks. The implementation of each callback is activated by a particular user action such as clicking a mouse button, pressing a screen button, typing a string or a numeric value, selecting a menu item, or passing the cursor over to a component. The GUI then reacts to these events. In this manuscript, GUI is used to construct and control the curves of the fractured segment of the parietal bone. The input for curve construction is missing part end points.

### Proposed Algorithm

This section simplifies the algorithm for craniofacial fracture reconstruction.

**Input**: CT scan image in Dicom format.

**Output**: Constructed 3D craniofacial fracture.

**Fig 7:**
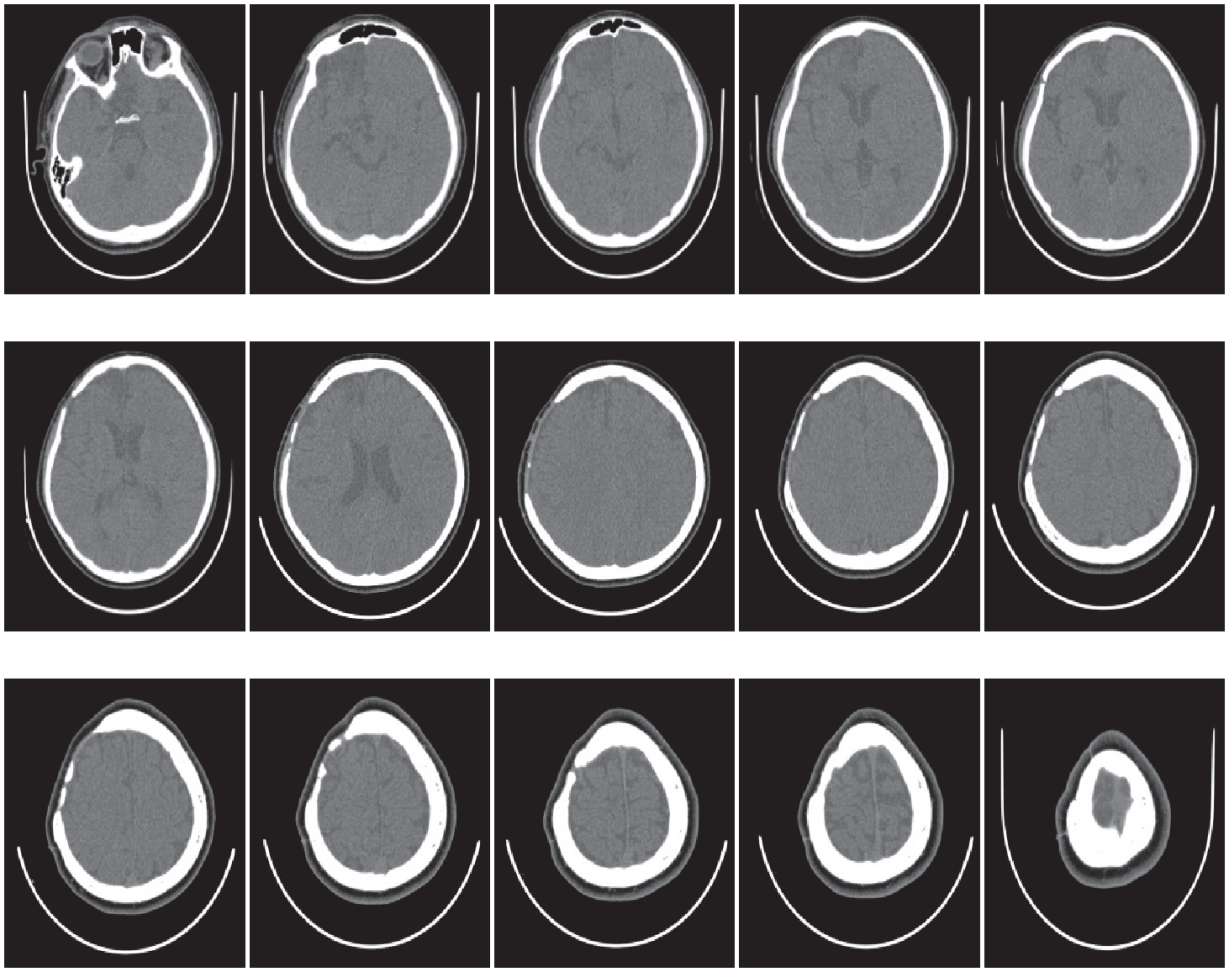
Given CT scanned images of patient with parietal bone fracture.

**Fig 8:**
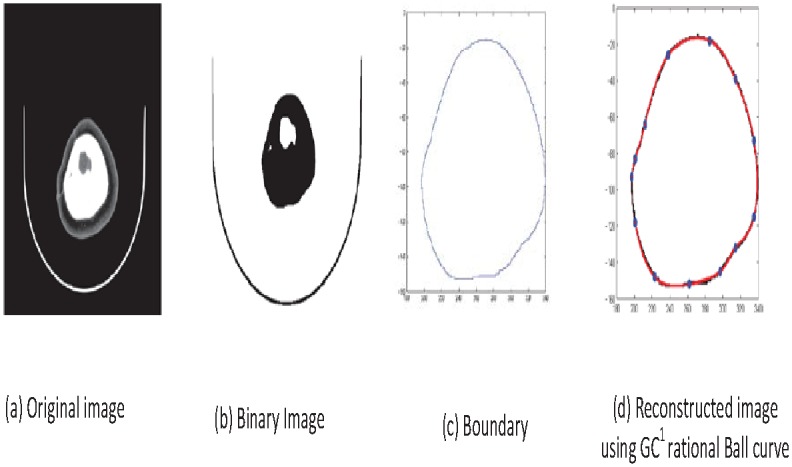
Reconstructed image of CT scan of Dicom slice 178.

**Fig 9:**
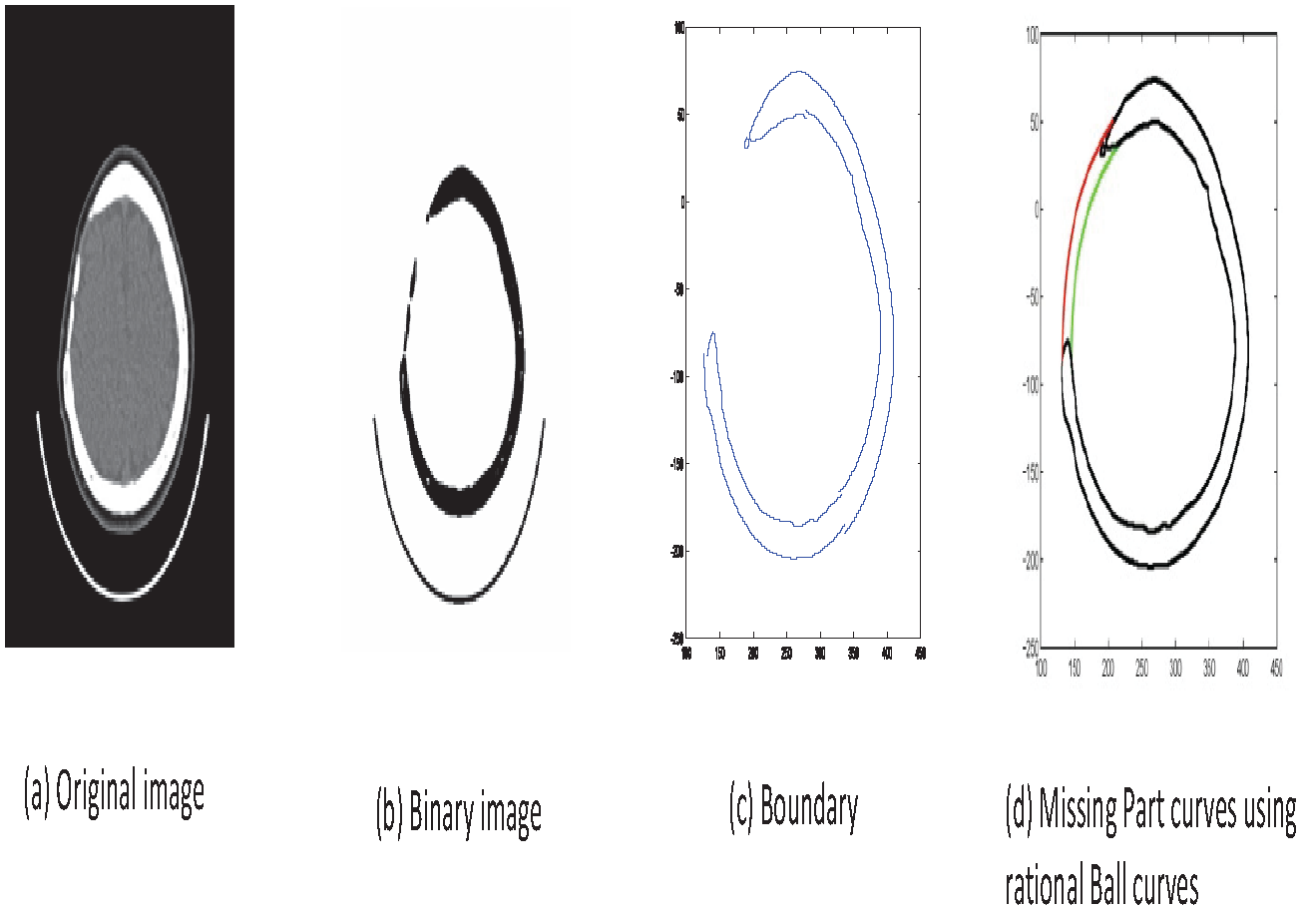
CT scanned data of slice 156.

**Fig 10:**
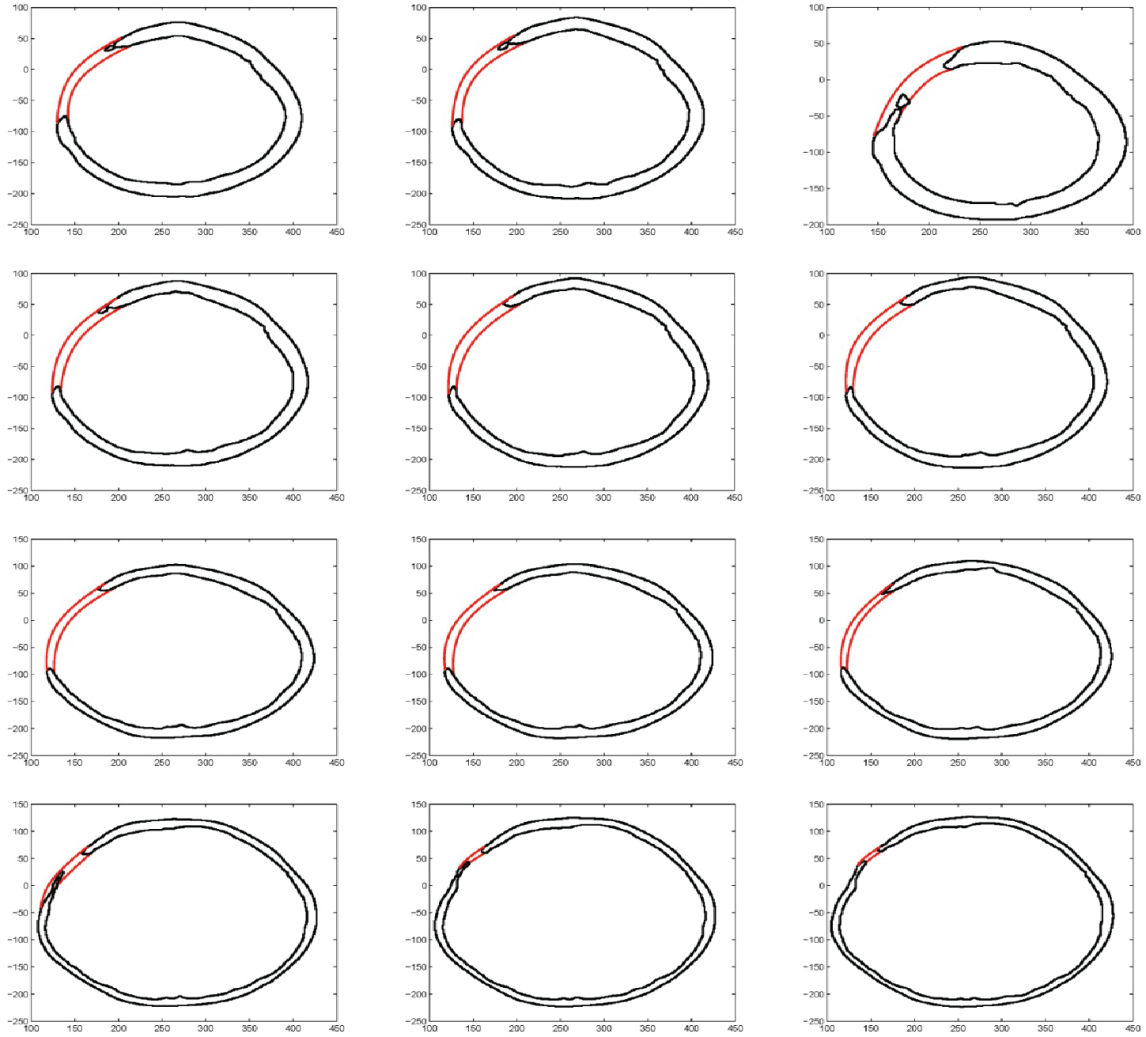
Reconstructed missing part curves for different CT scanned slices.

**Fig 11:**
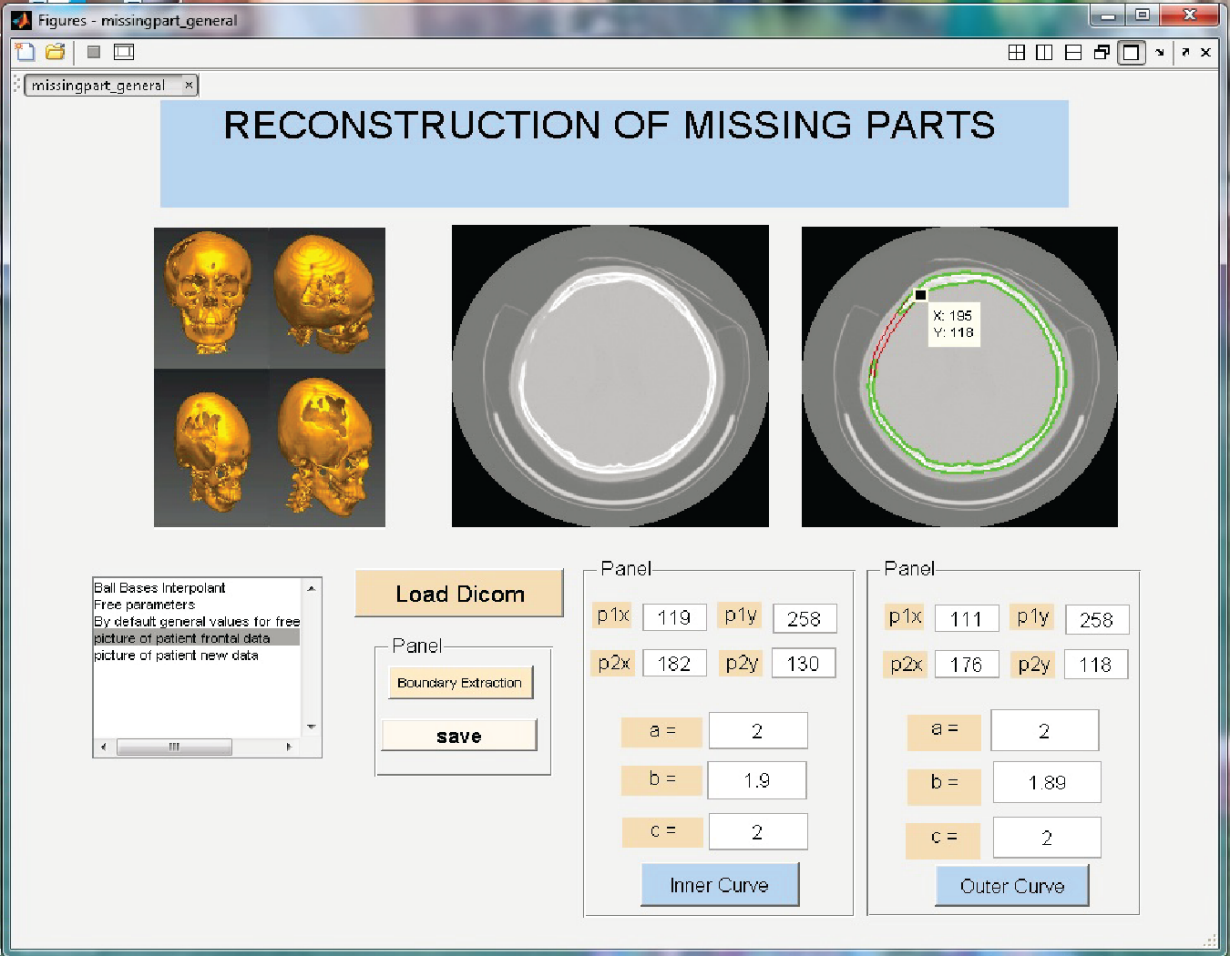
GUI display of craniofacial reconstruction.

**Fig 12:**
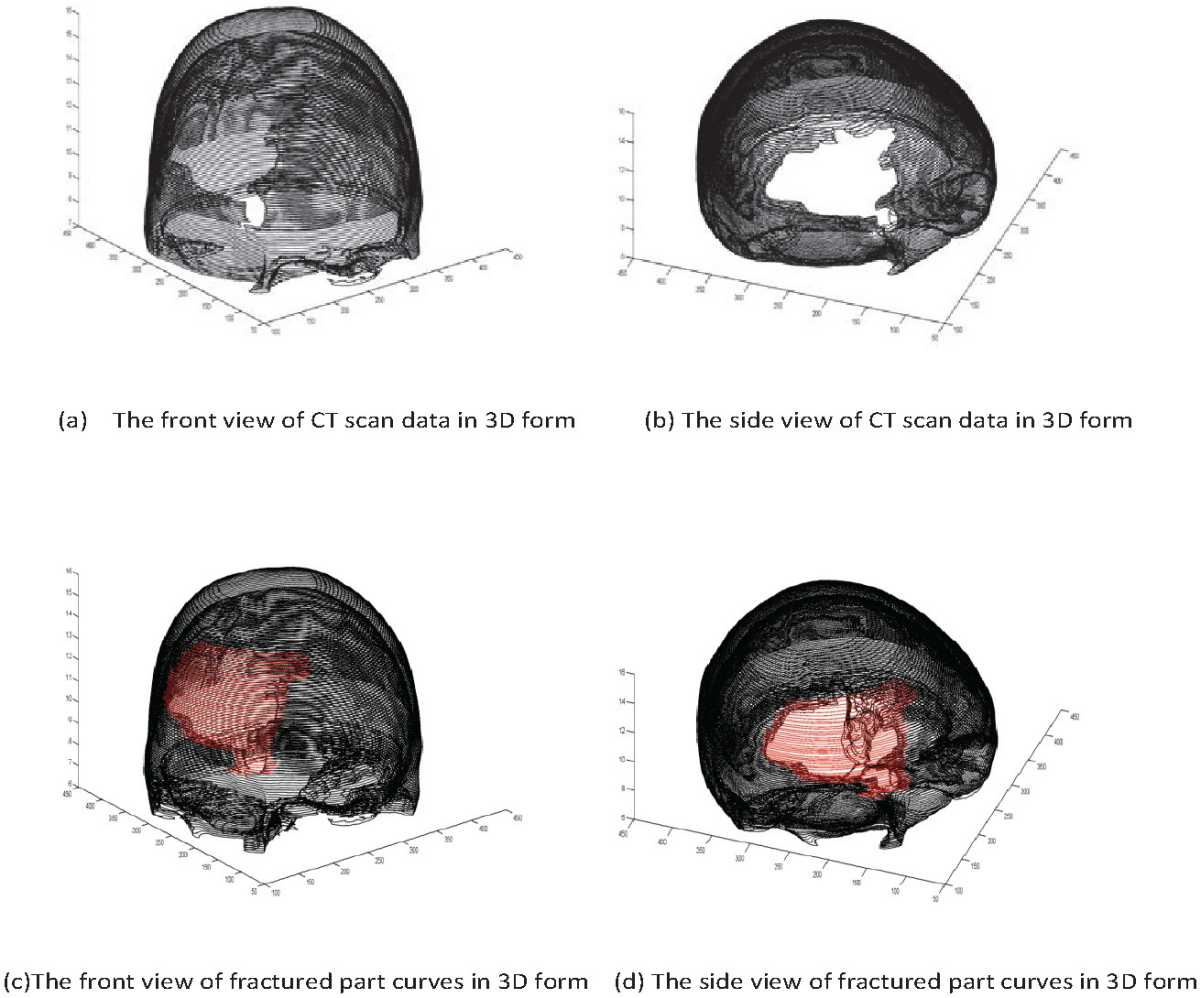
CT scan data in 3D format.

**Fig 13:**
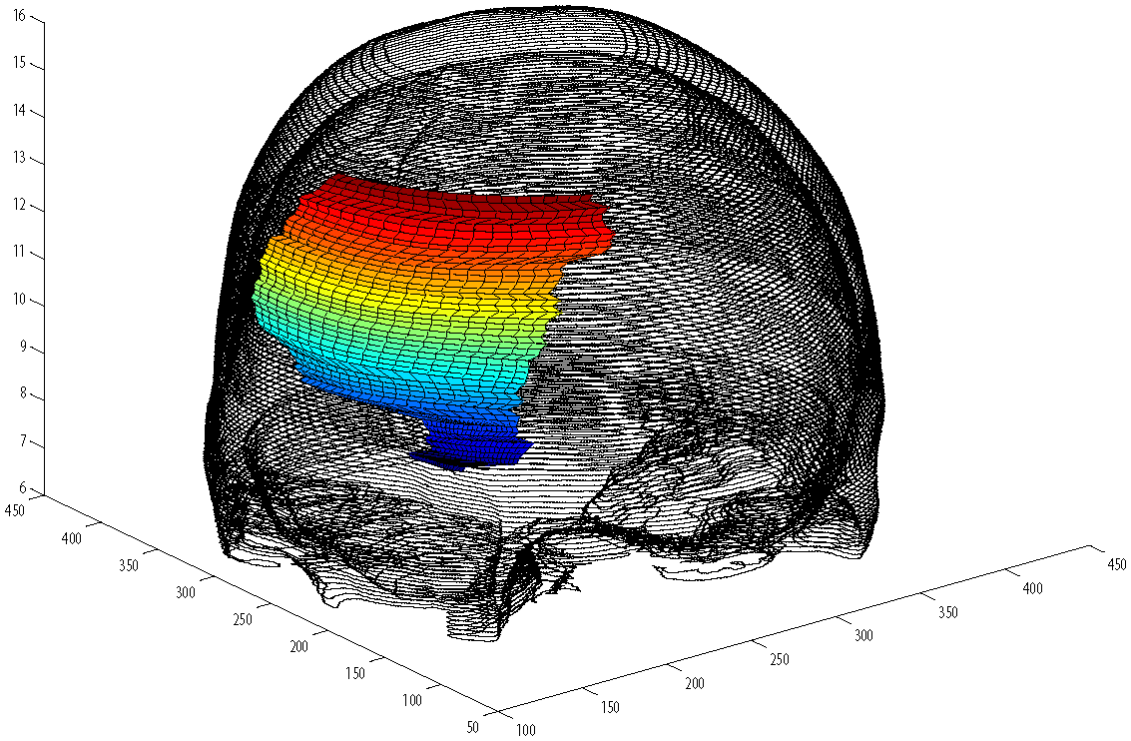
The front view of reconstructed 3D craniofacial fracture.

**Fig 14:**
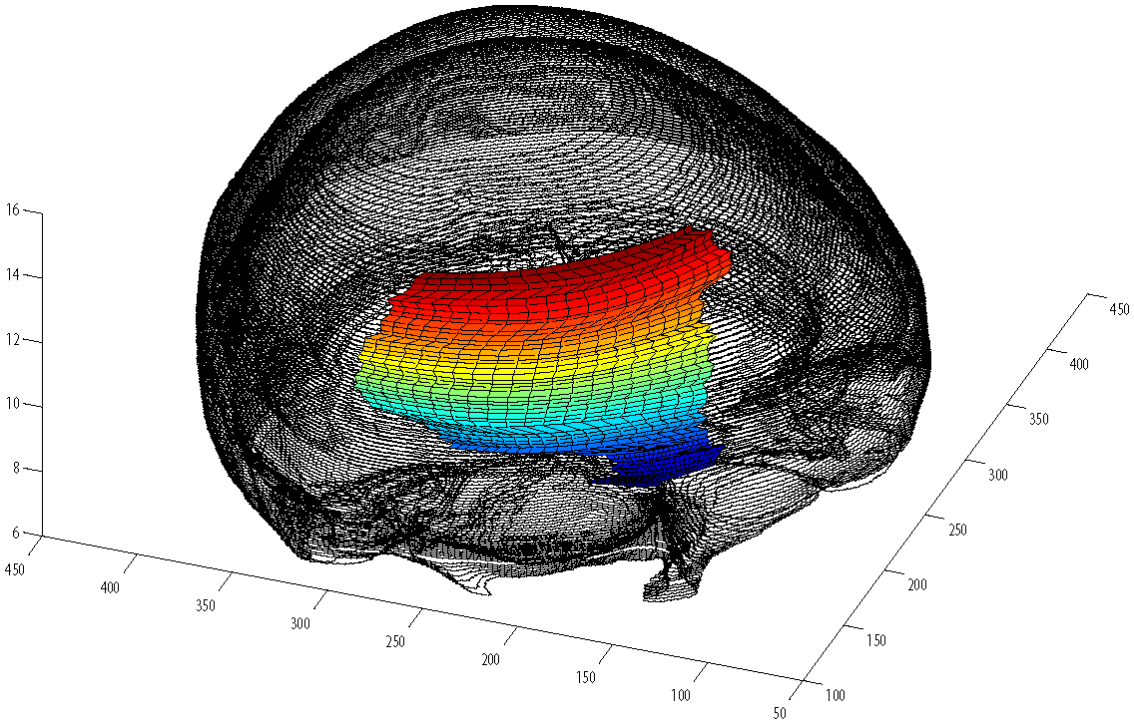
The side view of reconstructed 3D craniofacial fracture.

1. Read image as in Fig 7.
2. Boundary extraction Fig 8(c)
3. Corner points detection to make segments of curve Fig 8(d)
4. *GC*^1^ rational Ball curve is used to fit each segment. The unknown parameters *a*_*i*_, *b*_*i*_ and *λ*_*i*_ in Eq (2) are optimized using Genetic Algorithm Fig 8(d)
5. Step 4 is repeated until a desired solution is obtained
6. Fracture part curves reconstruction for each CT scan slice as in Figs 9 and 10
7. Using GUI for the construction of curves as in Fig 11
8. Conversion of 2D CT scan data into 3D form as in Fig 12
9. Construction of 3D craniofacial fracture using proposed method as in Figs 13 and 14.

In step 4 we reconstruct the boundary curve of a full skull using our proposed method. The genetic algorithm (GA) is used to optimize the free parameters. Normalized mean squares error is used as a cost function in GA and will be repeated until we get the best possible values of free parameters for boundary curve construction as shown in Fig 8(d). The process of optimization terminates when the error is less or equals to 10^−2^ for the desired solution. In Fig 8(d), reconstructed boundary curve is represented in red, while the original boundary curve is in black.

## Case study: 3D Craniofacial Fracture Reconstruction

This section illustrates an example of application of the proposed algorithm. The given 2D CT scan Dicom data are in slices as in Fig 7. First, we constructed the boundary curves of complete skull using rational cubic Ball with *GC*^1^ continuity at each knot as shown in Fig 8. Then, we have constructed the inner and outer curves of fractured part for all CT scan slices. Fig 9(a) is the original CT scan of slice 156. For the construction of fractured part, initially, we converted the original image to binary form as shown in Fig 9(b), then, using mathematical morphology, we extracted the boundary of skull as shown in Fig 9(c). Fig 9(d) represents the reconstructed inner and outer curves of fractured part using rational Ball curves. A least square method has been used to evaluate the intermediate control points of a rational Ball curve. The first and last control points of a rational Ball curve will be the starting and end point of the fractured part. The curves can be changed or alter using free parameters defined in Eq (6). Fig 10 shows the construction of fractured part curves of different slices. Fig 11 is the display of Graphical user interface (GUI) which helps in constructing the fractured curves. Next step is to convert the CT scan data and reconstructed fracture curves in 3D form taking equidistant *z* component as shown in Fig 12. Fig 12(a) and 12(b) are front and side view of CT scan data in 3D form respectively, similarly Fig 12(c) and 12(d) represent the front and side view of 3D CT scan data with fractured part curves respectively. Figs 13 and 14 represent the front and side view of constructed 3D parietal bone fracture using proposed contour blending function.

The Human Research Ethics Committee, Universiti Sains Malaysia, approved the study. Data were collected from archived images of patients from the Picture Archiving and Communication System (PACS) server at the Radiology Department, Hospital Universiti Sains Malaysia. The patients had undergone decompressive craniectomy surgery between May 2009 and December 2014 at the Hospital Universiti Sains Malaysia, Kubang Kerian, Kelantan Malaysia. The medical images were anonymized and the researchers had no access to patients’ information.

## Conclusion

Contours blending function have been used to construct the 3D surface. A patient with parietal bone fracture have been discussed as a case study to show its applicability. Fracture part curves of each contour is constructed in 2D form using *GC*^1^ rational Ball curves, since the given Dicom data are in 2D form. Then we convert each 2D contour into 3D form taking equidistant *z* component. The free parameters in proposed interpolant are optimized using Genetic Algorithm. The proposed method will provide the custom made implant for every individual patient. It is also time saving as the surgeon can perform the task of designing the implant geometry without the help of technicians and any other external help. It also reduces the incidence of infection. Proposed method is user friendly due to the presence of GUI.
